# Supplementary material for: A machine learning toolkit for genetic engineering attribution to facilitate biosecurity
Source: Nat Commun. 2020 Dec 8;11:6293. doi: 10.1038/s41467-020-19612-0 (PMC7722865; doi:10.1038/s41467-020-19612-0)
Supplement: Supplementary file 1 — Supplementary Information [file 41467_2020_19612_MOESM1_ESM.pdf]

Supplementary Information

Phenotype	Description	Example experiment to assay a sample organism	Possible Values	Number not missing	Number unique values
Bacterial Resistance(s)	Antibiotic resistance of the plasmid used for selecting during bacterial growth and cloning.	Expose the organism to concentration gradients of various antibiotics and measure growth.	Ampicillin, Kanamycin, Spectinomycin, Chloramphenicol, Other	67095	2189
Copy Number	Based on the Origin of Replication, the copy number is the number of plasmids per bacterial cell.	Quantitative PCR priming on target plasmid compared to known copy number	High, Low, Unknown	28517	490
Growth Strain	The strain used to clone the plasmid.	Various microbiological techniques; sequencing 16s and other markers	Dh5alpha, NEB stable, top10, stbl3, xl1 blue, DH10b, ccdb Survival	81717	200
Growth Temperature	The temperature the plasmid should be grown at.	Grow under temp. gradient and measure growth.	30 C, 37 C, Other	81717	91
Selectable Markers	For a plasmid used outside of the cloning organism, these markers allow non-bacterial selection.	As in the antibiotic case, but with other selection conditions.	Neomycin, Puromycin, Hygromycin, URA3, Blasticidin, Zeocin, Leu2, Trp1, His3, Other	81717	3

Species	The species the plasmid is used in, after cloning.	Various microbiological and taxonomic techniques; sequencing 16s and other markers	Human, Fly, Mouse, Budding Yeast, Zebrafish, Rat, Mustard Weed, Nematode, Other	81717	3

Supplementary Table 1. Simple phenotypic information inferred from Addgene. Note that in the envisioned deployment scenario some of these characteristics may be unavailable or uniformative, for example the growth strain used for cloning is not available if you have a sample of the chassis orgamism instead of cloning strain. On the other hand, many additional and more informative phenotypic characterizations are possible, and could be incorporated into the model if a sufficiently large and representative dataset of measurements and lab-of-origin are assembled.

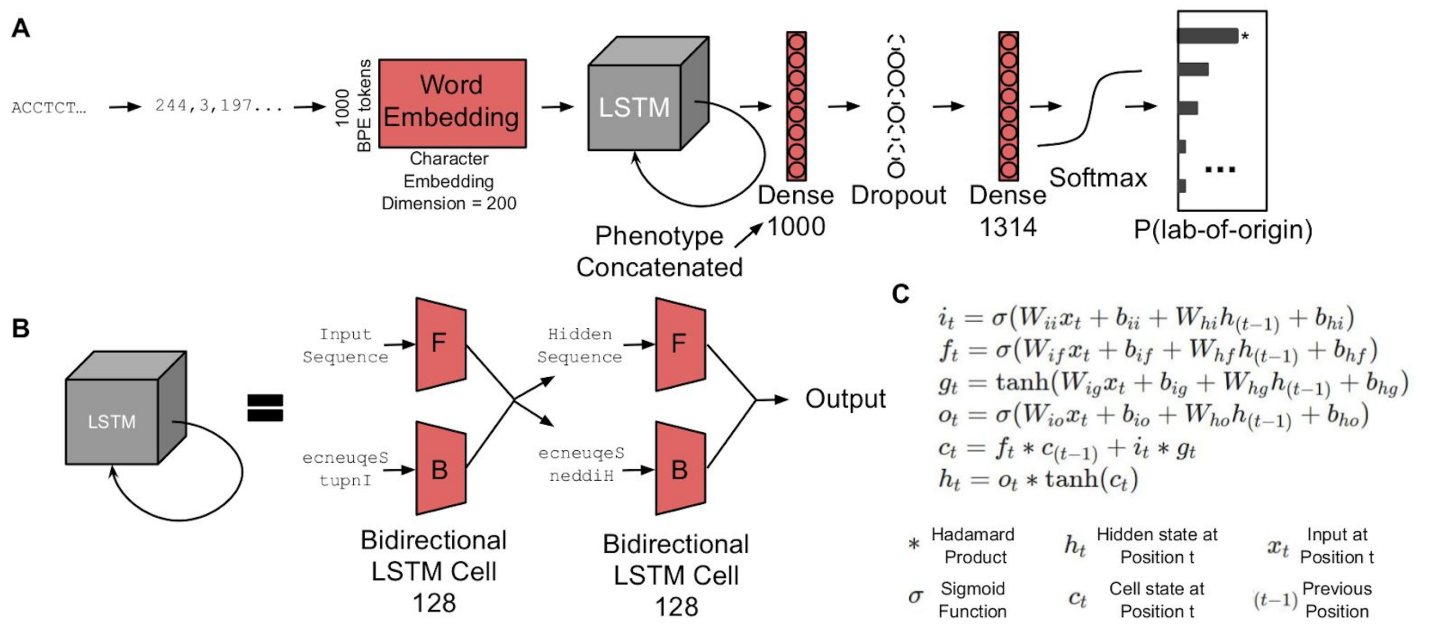

Supplementary Figure 1. Model architecture. (**A**) From left to right: a DNA sequence is Byte-Pair-Encoded to integers, embedded into a vector space, passed through a 2-layer bi-directional LSTM, to a 1000-d fully connected layer with dropout = .5, another fully connected layer which outputs 1314 dimensional logits, one for each class, which is softmaxed to produce a prediction vector summing to 1. (**B**) The LSTM. Input sequence is processed forward (F) and backward (B) by LSTM cells. The previous layer's hidden states are concatenated, and as before both the forward and backward sequence are processed. The output is the concatenation of the final hidden state from forward and backward cells. (**C**) Mathematical definition of an LSTM cell. At each position along a sequence, the output of the cell is defined by the value of the input sequence (x_t) and a recurrent relationship with the previous step, captured in a hidden state and cell state ($h_{(t-1)}$ $c_{(t-1)}$). Typically, i_t, f_t, g_t, o_t, are

called the input, forget, cell and output gates, respectively. For motivation and a full mathematical treatment, please see Hochreiter and Schmidhuber (1997)[59].

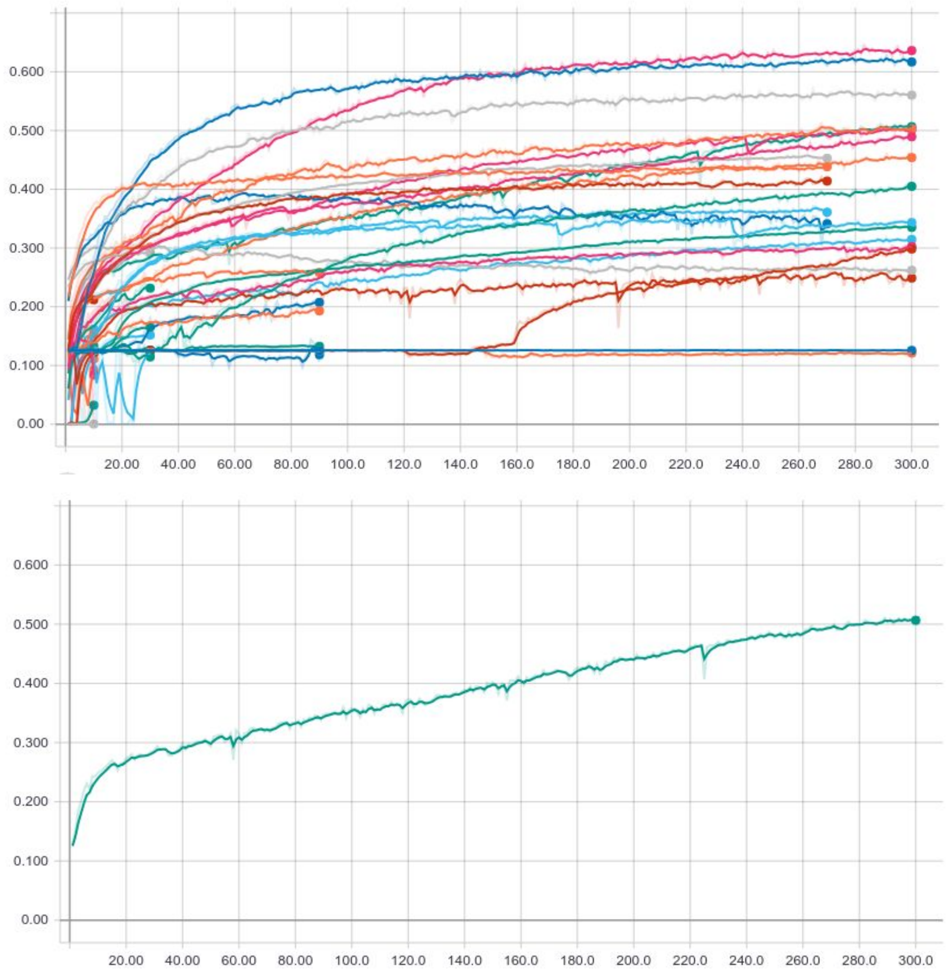

Supplementary Figure 2. Accuracy curves of Asynchronous Hyperband hyperparameter search. Validation accuracy on the Y axis, and number of Hyperband steps on the X axis. The entire collection of variants (top) is compared to the selected model (bottom).

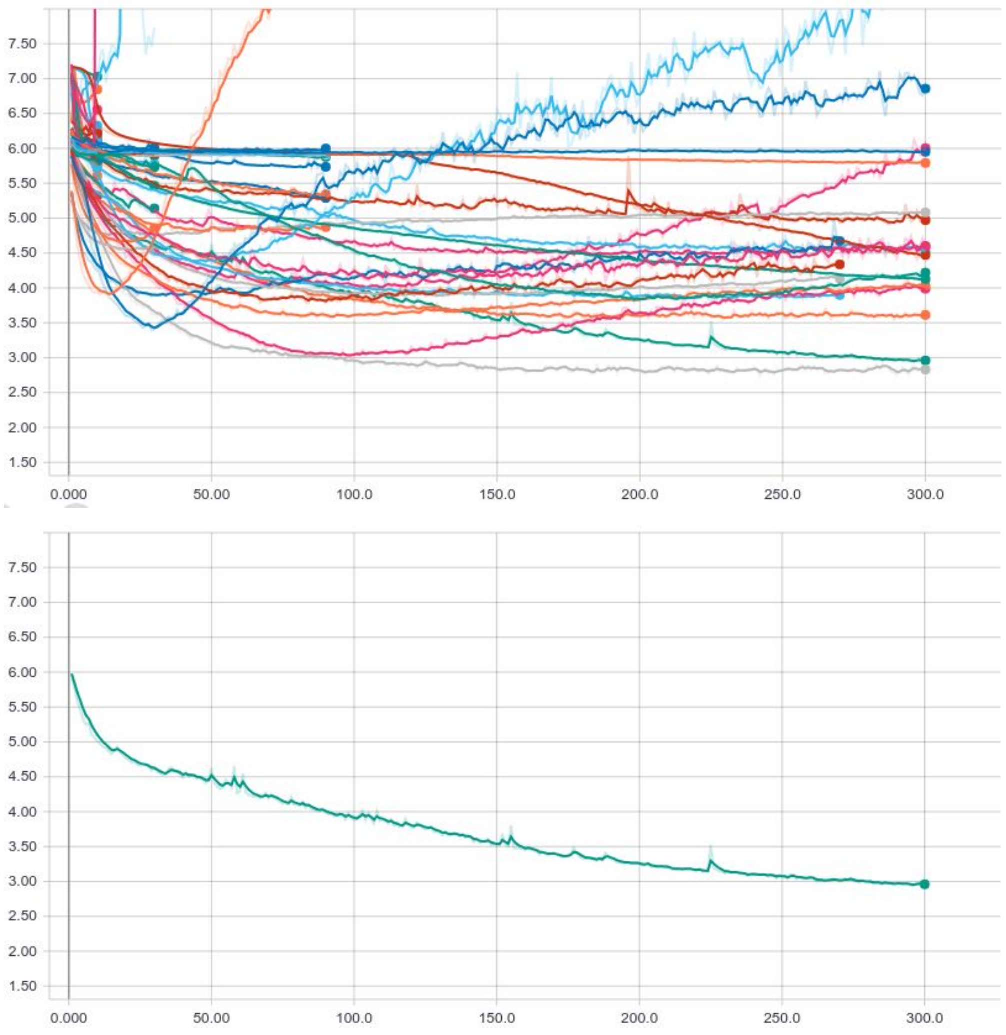

Supplementary Figure 3. Loss curves of Asynchronous Hyperband hyperparameter search. Validation set cross entropy loss on the Y axis, and number of Hyperband steps on the X axis. The entire collection of variants (top) is compared to the selected model (bottom).

Method	Top 1 Accuracy	Top 10 Accuracy
Ours	**70.1%**	**84.7%**
Ours -phenotype	59.9%	80.3%
BLAST	66.3%	74.8%
CNN (Nielsen & Voigt, 2018)	50.2%	73.4%
Baseline: guess by abundance in training set	7.5%	15.2%
Uniformly random guess	.076%	.76%

Supplementary Table 2. Lab-of-origin attribution accuracy on the held-out test set.

Method	Top 1 Accuracy	Top 10 Accuracy
Random Forest	**75.8%**	**96.7%**
BLAST	70.3%	86.7%
Baseline: guess by abundance in training set	16.1%	83.8%
Uniformly random guess	3.0%	30.3%

Supplementary Table 3. Nation-of-origin attribution accuracy on the held-out test set.

Method	Top 1 Accuracy	Top 10 Accuracy
Random Forest	**87.0%**	**96.5%**
Baseline: guess by abundance in training set	13.6%	45.0%
Uniformly random guess	.5%	5.3%

Supplementary Table 4. Ancestor lab attribution accuracy on the held-out test set.

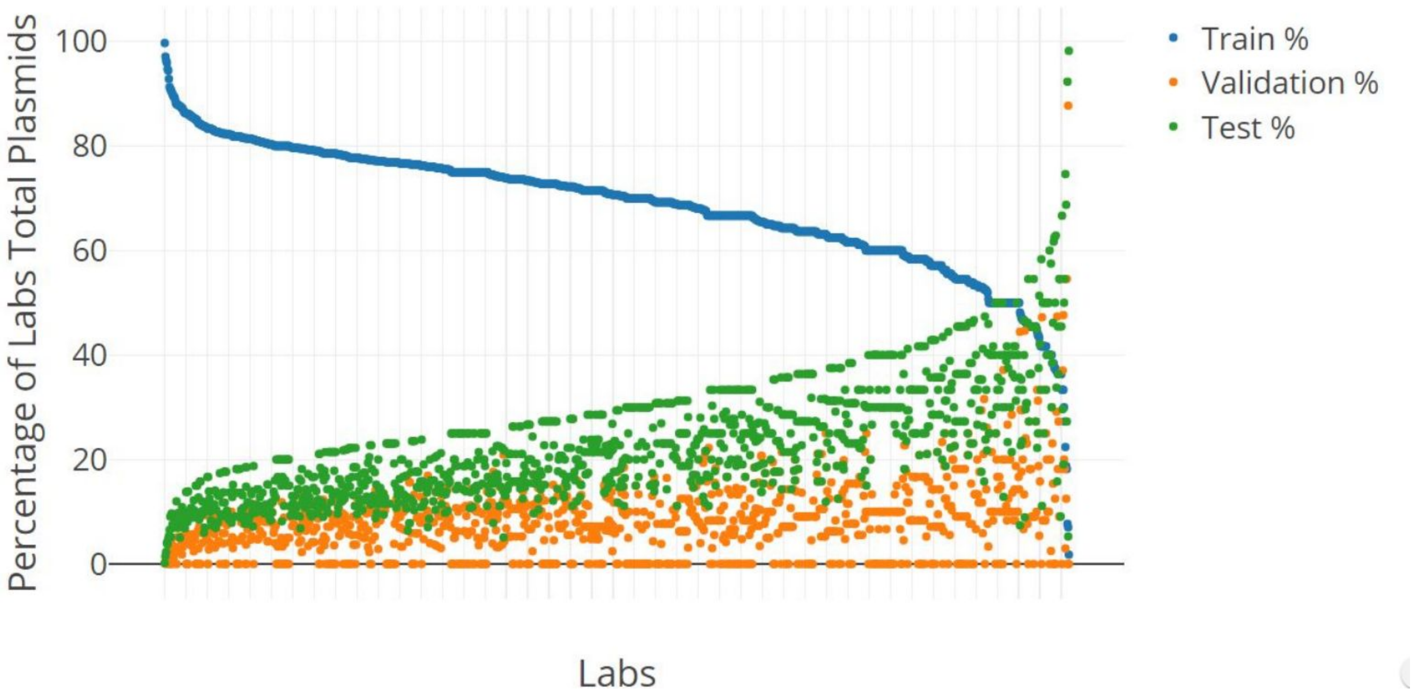

Supplementary Figure 4. Lab distribution after train-test-validation split. Each vertical sums to 100%. The validation set points (orange) hit 0% abundance because there was no rule that the validation set must have a certain number of plasmids per lab.

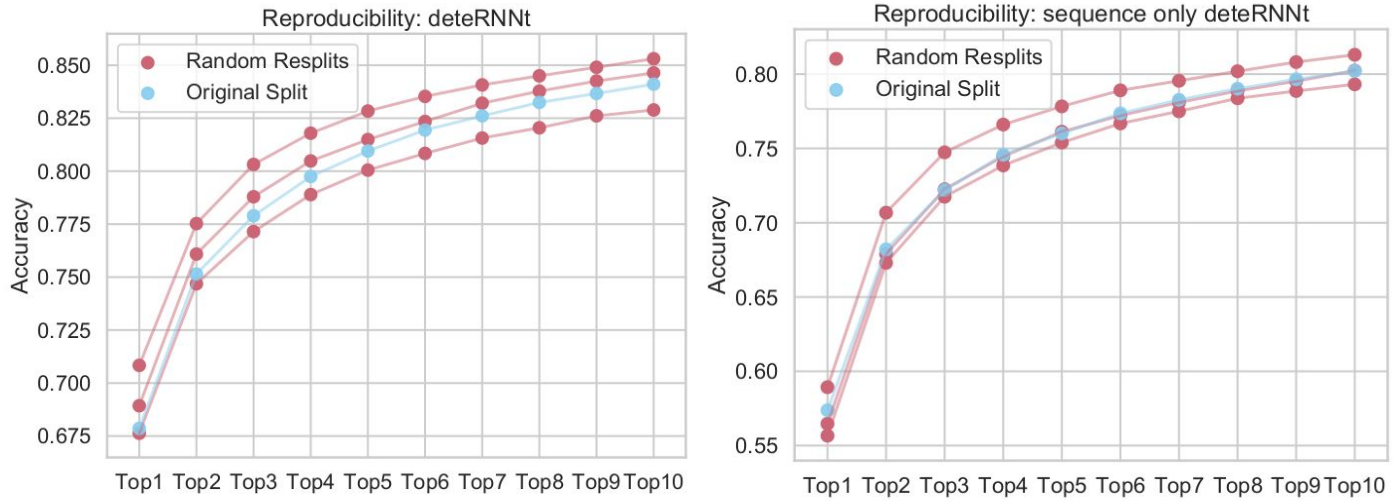

Supplementary Figure 5. Reproducibility of random data splits. Three full resplits of the data show on-par performance with the original split, using the same hyperparameters but different random seeds and input data. X axis shows TopN accuracy for N in [1-10]. Y axis shows test set accuracy for the splits corresponding test set. The original split (blue) has a different random seed and slightly different training time from the model presented in the main text but the same data.

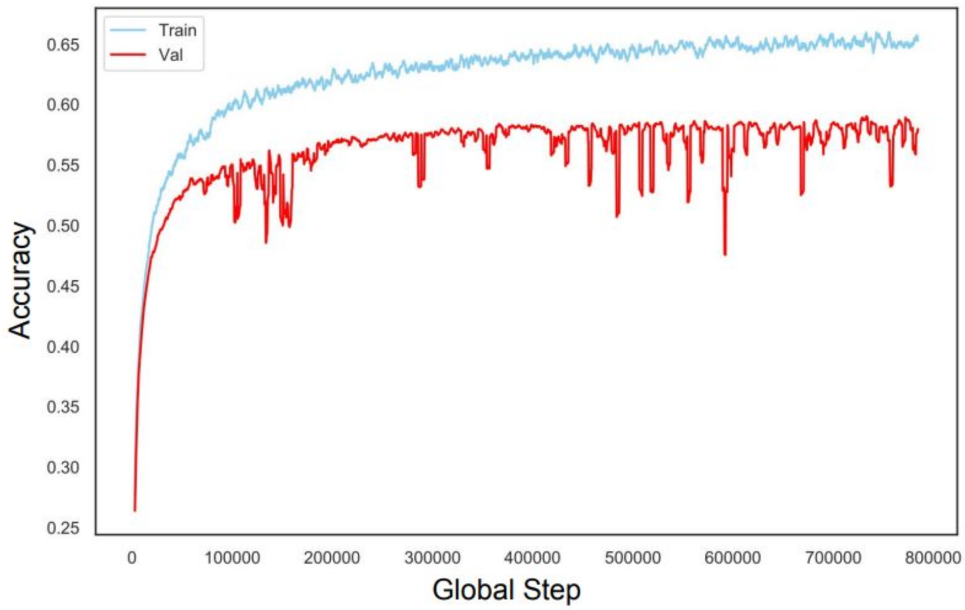

Supplementary Figure 6. Training and validation curves of the CNN model on Addgene lab-of-origin data. Training continues to 100 epochs, or 787,800 steps on our data.
