# Supplementary material for: A machine learning toolkit for genetic engineering attribution to facilitate biosecurity
Source: Nat Commun. 2020 Dec 8;11:6293. doi: 10.1038/s41467-020-19612-0 (PMC7722865; doi:10.1038/s41467-020-19612-0)
Supplement: Supplementary file 2 — Reporting Summary [file 41467_2020_19612_MOESM2_ESM.pdf]

nature research

Corresponding author(s): Ethan C. Alley

Last updated by author(s): Sep 24, 2020

Reporting Summary

Nature Research wishes to improve the reproducibility of the work that we publish. This form provides structure for consistency and transparency in reporting. For further information on Nature Research policies, see our Editorial Policies and the Editorial Policy Checklist.

Statistics

For all statistical analyses, confirm that the following items are present in the figure legend, table legend, main text, or Methods section.

n/a	Confirmed	
☐	☒	The exact sample size (n) for each experimental group/condition, given as a discrete number and unit of measurement
☒	☐	A statement on whether measurements were taken from distinct samples or whether the same sample was measured repeatedly
☐	☒	The statistical test(s) used AND whether they are one- or two-sided<br>*Only common tests should be described solely by name; describe more complex techniques in the Methods section.*
☐	☒	A description of all covariates tested
☐	☒	A description of any assumptions or corrections, such as tests of normality and adjustment for multiple comparisons
☐	☒	A full description of the statistical parameters including central tendency (e.g. means) or other basic estimates (e.g. regression coefficient) AND variation (e.g. standard deviation) or associated estimates of uncertainty (e.g. confidence intervals)
☐	☒	For null hypothesis testing, the test statistic (e.g. F, t, r) with confidence intervals, effect sizes, degrees of freedom and P value noted<br>*Give P values as exact values whenever suitable.*
☐	☒	For Bayesian analysis, information on the choice of priors and Markov chain Monte Carlo settings
☐	☒	For hierarchical and complex designs, identification of the appropriate level for tests and full reporting of outcomes
☐	☒	Estimates of effect sizes (e.g. Cohen's d, Pearson's r), indicating how they were calculated

Our web collection on statistics for biologists contains articles on many of the points above.

Software and code

Policy information about availability of computer code

Data collection	Data collection was performed with custom R (v 3.4.4) python (v3.6) and bash scripts. These are available on request.
Data analysis	Data analysis was performed with python (v 3.6) in jupyter notebooks and scripts, and is available at https://github.com/altLabs/attrib. In this repository a complete environment specification is provided with exact versions of every python package used as well as instructions to configure and install them. For components requiring a GPU, the exact ami for use on Amazon Web Services is also provided. Other software used in this work includes BLAST (v 2.8.1) and CHOPCHOP (v 3).

For manuscripts utilizing custom algorithms or software that are central to the research but not yet described in published literature, software must be made available to editors and reviewers. We strongly encourage code deposition in a community repository (e.g. GitHub). See the Nature Research guidelines for submitting code & software for further information.

Data

Policy information about availability of data

All manuscripts must include a data availability statement. This statement should provide the following information, where applicable:

- Accession codes, unique identifiers, or web links for publicly available datasets
- A list of figures that have associated raw data
- A description of any restrictions on data availability

The sequence of pCI-YFP sequence can be obtained from Genbank (Genbank JQ394803.1 [https://www.ncbi.nlm.nih.gov/nuccore/JQ394803.1]). pAAV-Syn-SomArchon is available on Addgene (Addgene #126941 [https://www.addgene.org/126941/]). AAEL010097-Cas9 is available on Addgene (Addgene #100707 [https://www.addgene.org/100707/]). U6a and U6c can be obtained from Addgene (Addgene #117221 [https://www.addgene.org/117221/] and #117223 [https://www.addgene.org/117223/], respectively). The sequence of the custom Akbari/ Boyden gene drive (Fig. 5 e) is available in the github repository associated with this paper at https://github.com/altLabs/attrib/blob/master/sequences/custom_drive.fasta. All other data are available on request.

Field-specific reporting

Please select the one below that is the best fit for your research. If you are not sure, read the appropriate sections before making your selection.

- [x] Life sciences
- [ ] Behavioural & social sciences
- [ ] Ecological, evolutionary & environmental sciences

For a reference copy of the document with all sections, see nature.com/documents/nr-reporting-summary-flat.pdf

Life sciences study design

All studies must disclose on these points even when the disclosure is negative.

Sample size	Sample size (e.g. number of replicates for bootstrap resampling p-value computations) were determined prospectively, based on the author's experience doing similar analyses for which the selected sample size was more than sufficient.
Data exclusions	As detailed in the methods, lab-of-origin classes with fewer than 10 examples had labels excluded and were merged into the unknown class. This was necessary because 10 examples is too few to both train and rigorously evaluate the generalization of a classification model. The decision to exclude these classes was made before any model training or prediction was attempted on the dataset.
Replication	The full deteRNNt training process was repeated successfully 3 times (Supp. fig 5) with replicate random splits after all other analyses were finalized. Top-k accuracies were on par with the deteRNNt model presented in the main test. Wherever else possible in the analysis, random seeds were fixed and notebooks were run multiple times to confirm reproducibility of analysis.
Randomization	Train-Validation-Test splits were randomly allocated when described as such, with additional striation criteria in the case of lab-of-origin to reduce co-occurrence of plasmids known to be derived from one another in the training and evaluation sets.
Blinding	Authors were blinded to validation set sequences when developing models. After early model prototyping, the validation set was then used to select the model and hyperparameters. The test set was kept hidden until the manuscript was being written. For end-to-end reproduction in Supp. Fig. 5, all other figures and text were completely finalized before re-splitting. We note that blinding was not enforced by a third party.

Reporting for specific materials, systems and methods

We require information from authors about some types of materials, experimental systems and methods used in many studies. Here, indicate whether each material, system or method listed is relevant to your study. If you are not sure if a list item applies to your research, read the appropriate section before selecting a response.

Materials & experimental systems

n/a	Involved in the study
x	☐ Antibodies
x	☐ Eukaryotic cell lines
x	☐ Palaeontology and archaeology
x	☐ Animals and other organisms
x	☐ Human research participants
x	☐ Clinical data
x	☐ Dual use research of concern

Methods

n/a	Involved in the study
x	☐ ChIP-seq
x	☐ Flow cytometry
x	☐ MRI-based neuroimaging
